# Comparison of the Effects of Fasting Glucose, Hemoglobin A_1c_, and Triglyceride–Glucose Index on Cardiovascular Events in Type 2 Diabetes Mellitus

**DOI:** 10.3390/nu11112838

**Published:** 2019-11-19

**Authors:** Wei-Yu Su, Szu-Chia Chen, Yu-Ting Huang, Jiun-Chi Huang, Pei-Yu Wu, Wei-Hao Hsu, Mei-Yueh Lee

**Affiliations:** 1 Department of General Medicine, Kaohsiung Medical University Hospital, Kaohsiung 807, Taiwan; s952135@gmail.com; 2 Division of Nephrology, Department of Internal Medicine, Kaohsiung Medical University Hospital, Kaohsiung Medical University, Kaohsiung 807, Taiwan; scarchenone@yahoo.com.tw (S.-C.C.); karajan77@gmail.com (J.-C.H.); wpuw17@gmail.com (P.-Y.W.); 3 Department of Internal Medicine, Kaohsiung Municipal Siaogang Hospital, Kaohsiung Medical University, Kaohsiung 812, Taiwan; my345677@yahoo.com.tw; 4 Faculty of Medicine, College of Medicine, Kaohsiung Medical University, Kaohsiung 807, Taiwan; 5 Division of Medical Statistics and Bioinformatics, Department of Medical Research, Kaohsiung Medical University Hospital, Kaohsiung Medical University, Kaohsiung 807, Taiwan; stakmuh@gmail.com; 6 Division of Endocrinology and Metabolism, Department of Internal Medicine, Kaohsiung Medical University Hospital, Kaohsiung Medical University, Kaohsiung 807, Taiwan

**Keywords:** fasting glucose, hemoglobin A_1c_, triglyceride-glucose index, cardiovascular events, type 2 diabetes mellitus

## Abstract

The triglyceride–glucose (TyG) index has been correlated with insulin resistance. We aim to investigate the role of the TyG index on cardiovascular (CV) events in type 2 diabetes mellitus and compare the roles of fasting glucose, hemoglobin A_1c_, and the TyG index in predicting CV events. This retrospective study enrolled 3524 patients with type 2 diabetes from the Kaohsiung Medical University Research Database in 2009 in this longitudinal study and followed them until 2015. The TyG index was calculated as log (fasting triglyceride level (mg/dL) × fasting glucose level (mg/dL)/2). CV events included myocardial infarction, unstable angina, stroke, hospitalization for coronary artery disease, peripheral artery disease, and CV-related death. The association between variables and CV events was assessed using a multivariable stepwise Cox proportional hazard analysis. Two hundred and fifteen CV events (6.1%) were recorded during a follow-up period of 5.93 years. The multivariable stepwise analysis showed that high fasting glucose (HR, 1.007; *p* < 0.001) and a high TyG index (HR, 1.521; *p* = 0.004) but not hemoglobin A_1c_ or triglycerides were associated with a higher rate of CV events. Adding fasting glucose and the TyG index to the basic model improved the predictive ability of progression to a CV event (*p* < 0.001 and *p* = 0.018, respectively), over that of hemoglobin A_1c_ (*p* = 0.084) and triglyceride (*p* = 0.221). Fasting glucose and the TyG index are useful parameters and stronger predictive factors than hemoglobin A_1c_ and triglyceride for CV events and may offer an additional prognostic benefit in patients with type 2 diabetes.

## 1. Introduction

It was estimated that there are 451 million people aged 18–99 years with diabetes worldwide in 2017; these numbers were expected to rise to 693 million by 2045. In 2017, approximately 374 million people with impaired glucose tolerance, and 5 million deaths worldwide were attributed to diabetes [1]. The risk of developing cardiovascular (CV) disease has been reported to be two- to three-fold higher in people with diabetes, in whom CV disease is the major cause of death [2,3]. Therefore, it is crucial to identify patients with type 2 DM at high risk of developing future CV events so that optimal management can be provided. Previous studies have demonstrated a progressive increase in the risk of CV events or death with increasing levels of fasting plasma glucose in both patients with and without diabetes [4,5]. Besides fasting glucose, hemoglobin A_1c_ is used as diagnostic criteria for type 2 DM. Several epidemiological studies have reported an association between hemoglobin A_1c_ and adverse CV outcomes [6]; others have not identified hemoglobin A_1c_ as a risk factor for adverse CV outcomes [7]. In a systematic review of 74 published studies and 46 studies of meta-analysis, hemoglobin A_1c_ is a dependable risk factor of overall and cardiovascular mortality in both diabetics and non-diabetics. An optimal hemoglobin A_1c_ level, ranging from 6.0% to 8.0% for people with diabetes and 5.0% to 6.0% for those without diabetes, results in the lowest all-cause and cardiovascular mortality [8].

High levels of triglycerides and fasting glucose are two components of the metabolic syndrome, which is one of the most important risk factors for CV disease [9]. The triglyceride-glucose (TyG) index combines both levels of triglycerides and fasting glucose, and it has been reported to be significantly correlated with insulin resistance and to be a reliable surrogate marker of insulin resistance [10]. Most previous studies have focused on the association between the TyG index and metabolic diseases [11,12,13], although several recent studies have shown an association between the TyG index and vascular disease [14,15] and CV outcomes in patients with non-ST-segment elevation acute coronary syndrome [16]. However, few studies have investigated an association between the TyG index and CV outcomes in patients with diabetes.

The aim of this study is to investigate whether the TyG index is associated with CV events in patients with type 2 DM and compare the roles of fasting glucose, hemoglobin A_1c,_ and the TyG index in predicting CV events. To the best of our knowledge, this is the first study to compare the roles of fasting glucose, hemoglobin A_1c,_ and the TyG index in predicting CV events in patients with type 2 DM.

## 2. Materials and Methods

### 2.1. Setting

Kaohsiung Medical University Hospital (KMUH) is a medical center located in southern Taiwan with around 1600 beds and 6000 patient visits per day. In this retrospective study, we used data from the KMUH research database (KMUHRD), which includes the data of approximately two million patients who attended KMUH from 2009 to 2015. The KMUHRD is comprised of data on hospital admissions, drug-dispensing records, ambulatory care, dental services, and biochemical test results. In addition, data on primary and secondary diagnoses coded according to the International Classification of Diseases (9th Revision, Clinical Modification; ICD-9-CM), dates of hospitalization, procedures, and discharge are recorded in the KMUHRD. The drug-dispensing data recorded in the KMUHRD include the type of prescriber, the name, date, amount, and prescribed dose regimen of the dispensed drug, and the length of the prescription (drug use period).

The KMUHRD is managed by the Division of Medical Statistics and Bioinformatics of KMUH. All personal identifiers are removed from data in the KMUHRD according to the Personal Information Protection Act in Taiwan and only authorized researchers are allowed to conduct data linkage, processing, and statistical analysis. Moreover, these researchers are required to use specific computers in a room with 24-h monitoring with encrypted identifiers, and they must also sign agreement forms. Furthermore, only tables and figures from the statistical analysis are permitted to be used after they have been inspected.

### 2.2. Study Population

**Figure 1:**
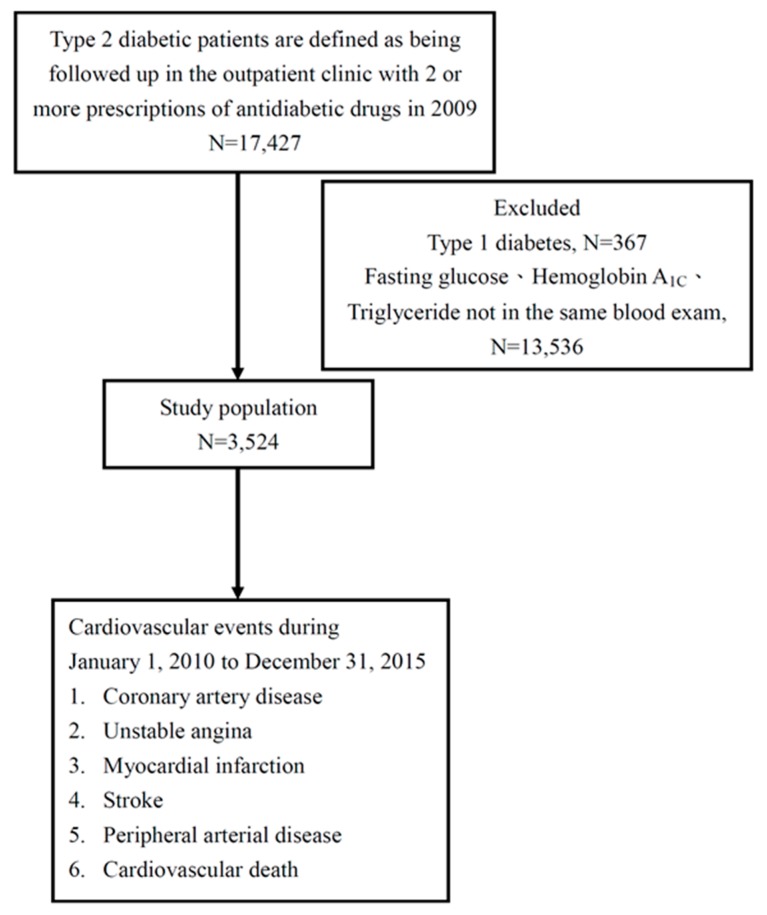
Flow chart of study participants for the evaluation of the effects of fasting glucose on cardiovascular events in type 2 diabetes.

All patients diagnosed with type 2 diabetes (ICD-9-CM codes 250.1–250.9) from 1 January 2009 to 31 December 2009, who were prescribed with hypoglycemic agents and who had a hemoglobin A_1c_ level ≥6.5% were enrolled and followed from 1 January 2010 to 31 December 2015. The covariates such as medications, comorbidities, age, measured were confirmed in 2009, which means if they used medications in 2009 or comorbidities according to ICD-9-CM code in 2009. In this study, CV events were defined as myocardial infarction (ICD-9-CM codes 410–412), coronary artery disease (ICD-9-CM code 414), unstable angina (ICD-9-CM code 411), ischemic stroke (ICD-9-CM codes 435–438), peripheral arterial disease (ICD-9-CM codes 443 and 25070) and CV death (ICD-9-CM code 785.51). Patients with type 1 diabetes, a hemoglobin A_1c_ level <6.5%, and those diagnosed with DM after the occurrence of a CV event were excluded (Figure 1).

In addition, the following data were recorded before entry into the study: age, sex, duration of diabetes, hyperlipidemia (ICD-9-CM code 272), hypertension (ICD-9-CM codes 401 and 405), retinopathy (ICD-9-CM code 250.50), nephropathy (ICD-9-CM codes 580–589), and neuropathy (ICD-9-CM codes 249.60 and 250.60).

### 2.3. Ethics Statement

The Institutional Review Board of KMUH approved this study (KMUHIRB-E(I)-20160032), and all of the patients provided written informed consent, including for the publication of clinical details. In addition, all clinical investigations were carried out according to the principles conveyed in the Declaration of Helsinki.

### 2.4. Definition of Study Endpoint

The study endpoint was defined as the occurrence of any CV event, including coronary artery disease, unstable angina, myocardial infarction, stroke, peripheral arterial disease, and CV-related death. Coronary artery disease was defined as ST- and non-ST-elevation myocardial infarction, history of angina, unstable angina, ischemic changes on electrocardiography, and having received angioplasty or coronary bypass surgery. In patients reaching study endpoints, data were censored at the CV events. The other patients were followed until death or December 2015.

### 2.5. TyG Index

The TyG index was calculated as log (fasting triglycerides (mg/dL) × fasting glucose (mg/dL)/2) [17], with at least three triglyceride and fasting glucose measurements, which product was the average of all blood draws in 2009. Laboratory data, including fasting glucose, triglyceride, and hemoglobin A_1c_, were measured from fasting blood samples.

### 2.6. Statistical Analysis

Data were expressed as percentages for categorical variables or mean ± standard deviation for continuous variables. Multivariable stepwise Cox proportional hazard analysis was used to evaluate associations between variables and CV events. Hemoglobin A_1c_ and significant variables in univariable analysis were used in the stepwise multivariable analysis. Associations among fasting glucose level, the TyG index, and CV events were assessed using three models. The first model included hemoglobin A_1c_ and significant variables in the univariable analysis except for the TyG index. The second model included hemoglobin A_1c_ and significant variables in the univariable analysis except for fasting glucose. Increases in model performance were assessed according to changes in the χ^2^ value. Statistical significance was set at *p* < 0.05. All statistical analyses were performed using SPSS 19.0 for Windows (SPSS Inc., Chicago, IL, USA).

## 3. Results

A total of 3524 patients (1731 males and 1793 females) with type 2 DM were included, and their clinical characteristics are shown in Table 1. Their mean age was 61.68 ± 11.90 years, and the average levels of fasting glucose and hemoglobin A_1C_ and TyG index were 154.83 ± 58.53 mg/dL, 7.85 ± 1.76%, and 9.09 ± 0.73, respectively.

### 3.1. Determinants of CV Events in the Study Patients

During the follow-up period of 5.93 ± 1.14 years, 215 CV events (6.1%) were recorded. Table 2 presents the univariable analysis of the factors associated with CV events in the study patients, which included old age, male sex, the prevalence of hypertension, neuropathy, nephropathy, coronary artery disease, peripheral artery disease, episodes of hypoglycemia, high fasting glucose, high triglycerides, low low-density lipoprotein (LDL) cholesterol, low estimated glomerular filtration rate (eGFR), high TyG index, and high urine albumin-creatinine ratio (UACR) were associated with increased CV events. In addition, medications including angiotensin-converting enzyme inhibitors and/or angiotensin II receptor blockers, anti-hypertensive drugs, aspirin, statins and/or fibrates, meglitinides, acarbose, and insulin were associated with increased CV events, whereas metformin use was associated with decreased CV events.

Table 3 shows the multivariable stepwise analysis of three models of the factors associated with CV events in the study patients. In multivariable stepwise Model 1, after adjusting for hemoglobin A_1c_ and significant variables in the univariable analysis (Table 2) except for the TyG index, old age, a history of coronary artery disease and peripheral artery disease, high fasting glucose (hazard ratio (HR), 1.007; 95% confidence interval (CI), 1.005 to 1.010; *p* < 0.001), low eGFR, high UACR, and the use of anti-hypertensive drugs and aspirin were independently associated with increased CV events. In multivariable stepwise Model 2, after adjusting for hemoglobin A_1c_ and significant variables in the univariable analysis (Table 2) except for fasting glucose, old age, a history of coronary artery disease, stroke and peripheral artery disease, a high TyG index (HR, 1.521; 95% CI, 1.141 to 2.027; *p* = 0.004), high UACR, and the use of anti-hypertensive drugs, aspirin, and insulin were independently associated with increased CV events. We further added the TyG index in Model 1 but did not change the results. Fasting glucose was significantly correlated with increased CV events, but the TyG index was not.

We have further performed subgroup analysis after excluding a history of CV events (*n* = 82) in Table 4, which shows the similar results. In Model 1, high fasting glucose (HR, 1.003; 95% CI, 1.001 to 1.005; *p* = 0.004), and a high TyG index (HR, 1.228; 95% CI, 1.015 to 1.486; *p* = 0.035) in Model 2 were associated with increased CV events.

### 3.2. Incremental Values of Fasting Glucose, Hemoglobin A_1c_, Triglyceride, and the Tyg Index in Relation to CV Events

**Figure 2:**
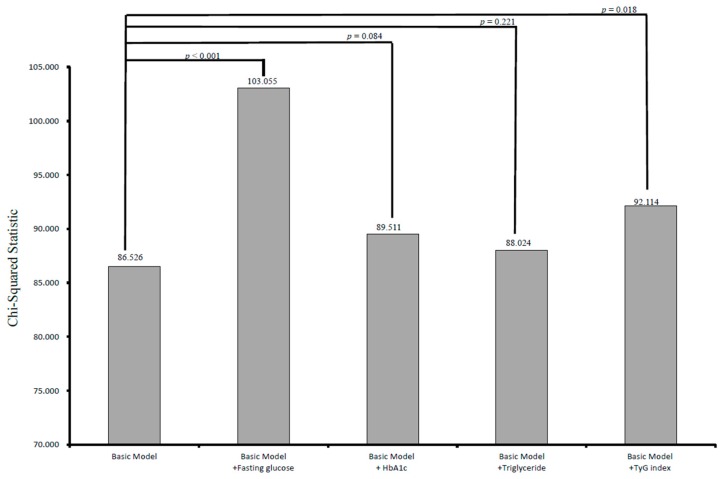
Comparison of the prediction power of addition of fasting glucose, hemoglobin A_1c_, triglyceride, and the TyG index to a basic model in the prediction of increased cardiovascular events. Addition of fasting glucose and TyG index resulted in a significant improvement in the prediction of increased cardiovascular events (*p* < 0.001 and *p* = 0.018, respectively), but hemoglobin A_1c_ and triglyceride (*p* = 0.084 and *p* = 0.221, respectively) did not.

The incremental values of fasting glucose, hemoglobin A_1c_, triglycerides, and the TyG index in outcome prediction are shown in Figure 2. The basic model included significant variables (Table 2) except for fasting glucose and the TyG index, including age, sex, the prevalence of hypertension, neuropathy, nephropathy, hypoglycemia episode, fasting glucose, triglycerides, LDL-cholesterol, eGFR, the TyG index, UACR, and the use of medications (χ^2^ = 86.526). Adding fasting glucose to the basic model improved the predictive ability of progression to CV events (χ^2^ = 103.055, *p* < 0.001). In addition, adding the TyG index to the basic model resulted in a significant improvement in the prediction of CV events (χ^2^ = 92.114, *p* = 0.018). However, adding hemoglobin A_1c_ and triglycerides to the basic model did not significantly improve the prediction of CV events (χ^2^ = 89.511, *p* = 0.084; χ^2^ = 88.024, *p* = 0.221, respectively).

## 4. Discussion

This study investigated the role of the TyG index in predicting CV events and compared the roles of fasting glucose, hemoglobin A_1c,_ and the TyG index in predicting CV events in patients with type 2 DM over a follow-up period of 5.93 years. The results showed that fasting glucose and the TyG index were associated with increased CV events, but that hemoglobin A_1c_ was not associated with increased CV events. Furthermore, our results showed that fasting glucose and the TyG index may improve the prognostic ability in patients with type 2 diabetes.

The first important finding in this study is that a higher level of fasting glucose was associated with an increased risk of CV events in the patients with DM and that it offered an additional prognostic benefit. An increasing number of studies have shown that abnormal glucose metabolism can accelerate the formation of atherosclerotic plaque, contributes to plaque rupture and thrombosis, and impair normal endothelial function. [18]. As mentioned in the Rotterdam Study which included elderly participants with a fasting blood glucose <110 mg/dL, those with higher blood glucose levels had higher levels of arterial stiffness [19]. Moreover, arterial endothelial dysfunction and intima-media thickening were associated with higher levels of glycemia (102–124 mg/dL) in the Chinese Atherosclerosis in the Aged and Young (CATHAY) study, [20], and Andreozzi et al. identified positive dose–response relationships between various CV disease biomarkers and fasting glucose levels [21]. Furthermore, in a study with a large multiethnic cohort, Anand et al. reported that the risk of CV events or death increased by 17% for each 1 mmol/L increase in fasting plasma glucose among individuals who were normoglycemic, those who had impaired fasting glucose or impaired glucose tolerance, and those newly diagnosed with diabetes [4]. In a meta-regression analysis of published data from 20 studies of 95,783 individuals followed for 12.4 years (range: 4–19 years), compared with a glucose level of 4.2 mmol/L (75 mg/dL), fasting and 2-h glucose levels of 6.1 mmol/dL (110 mg/dL) and 7.8 mmol/l (140 mg/dL) were associated with relative CV event risks of 1.33 and 1.58, respectively [5]. The results from the present study provide additional evidence of an association between an increased fasting plasma glucose level and the risk of CV events in patients with type 2 DM.

The second important finding in this study is that the TyG index was also associated with an increased risk of CV events and that it offered an additional prognostic benefit in predicting CV events in patients with type 2 diabetes. The TyG index includes both triglycerides and fasting glucose, and it has been demonstrated to be a good marker of insulin resistance and a predictor of type 2 DM [11,13]. In Korean adults, the TyG index has been associated with the progression of coronary artery calcification, which is considered to be a surrogate marker to predict the risk of CV disease [22]. In addition, a higher TyG index was significantly associated with an increased risk of developing CV disease in the vascular, metabolic CUN (VMCUN) cohort [23]. Other studies on the relationship between the TyG index and CV disease have also been reported. Sánchez-Íñigo et al. [23] reported a significant association between the TyG index and a high risk of developing CV disease in the VMCUN cohort, and that it was a good predictor for the Framingham model in these patients. However, Vega et al. [24] reported that the TyG index could only predict type 2 diabetes rather than CV disease compared to the ratio of triglycerides to high-density lipoprotein cholesterol. In addition, Mao et al. [16] investigated associations between the TyG index and CV outcomes in patients with non-ST-segment elevation acute coronary syndrome and found increased prevalence rates of glucose metabolism disorders, metabolic syndrome, and major adverse CV events with an increase in the TyG index. Pathophysiologically, insulin resistance is caused by GLUT4 reducing glycogen synthesis in skeletal muscles and is also influenced by circulating fatty acid levels. This oxidation impairment and utilization of fatty acids results in the flow of free fatty acids from adipose to non-adipose tissues and worsens many of the basic metabolic derangements that characterize insulin resistance, thereby increasing the risk of CV disease [23,25,26]. Insulin resistance as estimated by the homeostasis model assessment index (HOMA-index) has been shown to be able to predict the progression of atherosclerotic plaques in patients with coronary heart disease both with and without diabetes [27]. Furthermore, prospective data from the Verona Diabetes Complications Study showed that the HOMA index is an independent predictor of CV disease in patients with type 2 diabetes [28].

The third important finding of this study is that an increased level of hemoglobin A_1c_ was not associated with increased CV events in our cohort of diabetic patients. This finding is consistent with a previous study that showed no association between hemoglobin A_1c_ and the risk of new CV events during follow-up [7]. Trials using intensive blood glucose control have not shown to improved CV disease risk in populations with type 2 DM; however, in the general population, there are inconsistent reports about the effects of maintaining lower glucose levels. Some may assume that low glycemic values are associated with increased glycemic variability, which is in turn is associated with higher CV disease risk. It has also been proposed that fasting glucose and hemoglobin A_1c_ in the lower levels have a different relationship with CV disease and mortality. The Action in Diabetes and Vascular Disease: Preterax and Diamicron MR Controlled Evaluation (ADVANCE) study reported that visit-to-visit variability in both hemoglobin A_1c_ and fasting glucose could predict the future development of macrovascular and microvascular events and all-cause mortality. Furthermore, increases in the variability of hemoglobin A_1c_ and fasting glucose in the first two years were associated with increased risks of subsequent vascular events and mortality in diabetic patients [29]. In the Multi-Ethnic Study of Atherosclerosis (MESA) study, 4990 participants with low baseline glucose and hemoglobin A_1c_ were positively associated with mortality, whereas low mean fasting glucose and hemoglobin A_1c_ were significantly associated with the incidence of CV disease and mortality. Glucose variability, however, did not explain CV disease risk beyond the mean glucose levels, but long-standing low fasting glucose and hemoglobin A_1c_ may be better indicators of risk rather than a single low measurement [30]. Recent studies also mentioned the J-shaped association between hemoglobin A_1c_ and the risk of all-cause mortality among men and women with type 2 diabetes. Both high and low levels of hemoglobin A_1c_ were associated with an increased risk of all-cause mortality [31,32]. These findings are similar to those of another study using the Veterans Affairs Diabetes Trial (VADT), which reported that variability in fasting glucose was significantly associated with CV disease in patients receiving intensive glycemic control even after adjusting for other risk factors. However, the level of hemoglobin A_1c_ was not associated with CV disease after adjusting for multiple baseline risk factors [33]. Although findings of increased predictive value with visit-to-visit glucose variability with regards to the complications of diabetes have been inconclusive [33,34,35], a prior review and meta-analysis reported positive associations between hemoglobin A_1c_ variability and adverse outcomes including diabetic retinopathy and neuropathy, renal disease, CV macrovascular events, and death in patients with diabetes [36].

The fourth important finding of this study is that after multivariable stepwise analysis, a high level of triglycerides was not associated with increased CV events. In a prospective cohort of women in the United States who were healthy, it was initially observed that higher non-fasting triglyceride levels were strongly associated with an increased risk of future CV events [26]. In Asian populations, a recent systemic review found that triglyceride is an independent but significant risk factor for coronary heart disease, but is not a significant risk factor for stroke or peripheral arterial disease [37]. The Fenofibrate Intervention and Event Lowering in Diabetes (FIELD) trial was designed to evaluate the effect of fenofibrate (*n* = 4895) compared to placebo (*n* = 4900) on CV events over a 5-year follow-up period among men and women (50~75 years of age) with type 2 DM [38]. The results revealed that plasma triglycerides, LDL-cholesterol, and high-density lipoprotein cholesterol levels responded favorably to fenofibrate treatment, but that there was no significant difference between the treatment groups in the primary outcome of coronary events (coronary heart disease, death, or nonfatal myocardial infarction). In addition, there was a non-significant reduction of 11% between the patients receiving fenofibrate compared to those receiving a placebo, which corresponded to a significant 24% reduction in nonfatal myocardial infarction and a non-significant increase in coronary heart disease mortality. Whether triglycerides are an independent risk factor for coronary artery disease remains controversial.

Another important finding of this study is that fasting glucose and the TyG index were significantly associated with increased CV events in Model 1 and 2 (Table 3), but that triglycerides were not. Further, we added the TyG index in Model 1 and found that fasting glucose was significantly correlated with increased CV events, but that the TyG index was not. Moreover, the predictive power of fasting glucose for CV events was stronger than the TyG index, which is influenced by both fasting glucose and triglycerides. Therefore, our results indicate that adding triglycerides as a surrogate marker may weaken the relationship between the TyG index and CV events. Thus, we suggest that fasting glucose is the most important marker to predict the risk of CV events in patients with type 2 DM.

Limited studies have explored the role of the TyG index on CV outcomes in diabetes. The strength in this study is that it is the first study to investigate the comparison of the effects between fasting glucose, hemoglobin A_1c_, and the TyG index on CV events in a large number of patients with type 2 DM over a follow-up period of 5.93 years. There are several limitations to this study. First, this was an observational study, and therefore there were individual variations in the number and frequency of triglyceride, hemoglobin A_1c_, and fasting glucose measurements. To minimize this, patients who were followed for <6 months and had fewer than three measurements during the follow-up period were excluded. Second, cardiac autonomic neuropathy has been reported to potentially be a predisposing factor for CV events [39]. However, we did not evaluate heart rate variability in this study, and therefore could not analyze the effect of cardiac autonomic neuropathy on CV events, although we did include a diagnosis of diabetic neuropathy (according to ICD-9-CM code) in the analysis. Third, we validated these comorbidities using ICD-9-CM code. However, if individuals with type 2 DM visited the KMUH, but ultimately were diagnosed with a CV event or condition at another location, this would cause an underestimation of the disease risk. In addition, we did not calculate the insulin resistance index with the HOMA index, so we could not compare the role of the TyG index and HOMA-index in this study. Lastly, we did not evaluate the effect of medications on CV events, as this was not the aim of the study. The positive association between the use of medications and CV events may be due to selection bias.

## 5. Conclusions

In conclusion, higher fasting glucose and a high TyG index were associated with an increased risk of CV events among our study cohort with type 2 DM, but hemoglobin A_1c_ was not. Adding fasting glucose and the TyG index to the basic model offered an additional benefit in the prediction of progression to CV events. These findings support the potential role of fasting glucose and the TyG index as a major predictor of CV events, independently of hemoglobin A_1c_, in patients with type 2 DM. Assessments of fasting glucose and TyG index may be beneficial to allow for early stratification and interventions to prevent CV events.

## Figures and Tables

**Table 1:** Clinical characteristics of the study patients.

Characteristics	All (*n* = 3524)
Age (year)	61.68 ± 11.90
Male gender (%)	49.1
Hypertension (%)	69.7
Dyslipidemia (%)	69.8
Retinopathy (%)	5.4
Neuropathy (%)	13.4
Nephropathy (%)	5.0
Coronary artery disease (%)	1.8
Stroke (%)	0.4
Peripheral artery disease (%)	0.1
DM duration > 5 years (%)	87.5
Hypoglycemia episode (%)	3.6
Laboratory parameters	
Fasting glucose (mg/dL)	154.83 ± 58.53
Hemoglobin A1c (%)	7.85 ± 1.76
Triglyceride (mg/dL)	150.8 ± 217.81
Total cholesterol (mg/dL)	179.37 ± 44.00
HDL-cholesterol (mg/dL)	41.16 ± 13.04
LDL-cholesterol (mg/dL)	104.08 ± 33.86
eGFR (mL/min/1.73 m2)	87.66 ± 35.89
TyG index	9.09 ± 0.73
UACR (mg/g)	149.37 ± 531.69
Medications	
ACEI and/or ARB use (%)	65.6
Anti-hypertensive drugs use (%)	53.6
Aspirin use (%)	33.2
Statin and/or fibrate use (%)	70.7
Sulfonyurea use (%)	72.8
Metformin use (%)	82.2
Meglitinides use (%)	13.3
Pioglitazone use (%)	36.3
Acarbose use (%)	20.4
DPP-4 inhibitor use (%)	34.4
Insulin use (%)	25.9
CV events (%)	6.1
Follow-up time (year)	5.93 ± 1.14

Abbreviations: DM, diabetes mellitus; HDL, high-density lipoprotein; LDL, low-density lipoprotein; eGFR, estimated glomerular filtration rate; TyG, triglyceride-glucose; UACR, urine albumin-creatinine ratio; ACEI, angiotensin-converting enzyme inhibitor; ARB, angiotensin II receptor blocker; DPP-4, Dipeptidyl peptidase-4 inhibitor; CV, cardiovascular. The TyG index was calculated as log (fasting triglyceride (mg/dL) × fasting glucose (mg/dL)/2).

**Table 2:** Determinants for cardiovascular events using the Cox proportional hazards model (univariable analysis).

Parameters	Univariable	
	HR (95% CI)	*p*
Age (per 1 year)	1.035 (1.022–1.047)	<0.001
Male gender	1.422 (1.084–1.864)	0.011
Hypertension	2.435 (1.680–3.528)	<0.001
Dyslipidemia	1.057 (0.786–1.420)	0.715
Retinopathy	1.615 (0.997–2.618)	0.052
Neuropathy	1.425 (1.004–2.023)	0.048
Nephropathy	1.924 (1.201–3.082)	0.007
Coronary artery disease	8.524 (5.528–13.144)	<0.001
Stroke	2.924 (0.727–11.767)	0.131
Peripheral artery disease	17.399 (5.566–54.390)	<0.001
DM duration > 5 years	1.508 (0.930–2.445)	0.096
Hypoglycemia episode	2.238 (1.345–3.724)	0.002
Laboratory parameters		
Fasting glucose (per 1 mg/dL)	1.003 (1.001–1.005)	0.001
Hemoglobin A1c (per 1%)	1.069 (0.996–1.146)	0.064
Triglyceride (log per 1 mg/dL)	1.387 (1.125–1.709)	0.002
Total cholesterol (per 1 mg/dL)	1.001 (0.999–1.004)	0.310
HDL-cholesterol (per 1 mg/dL)	0.971 (0.958–0.983)	<0.001
LDL-cholesterol (per 1 mg/dL)	0.999 (0.995–1.004)	0.776
eGFR (per 1 mL/min/1.73 m^2^)	0.979 (0.975–0.984)	<0.001
TyG index (per 1)	1.342 (1.136–1.586)	<0.001
UACR (per 10 mg/g)	1.005 (1.003–1.006)	<0.001
Medications		
ACEI and/or ARB use	2.838 (1.967–4.094)	<0.001
Anti-hypertensive drugs use	3.774 (2.685–5.304)	<0.001
Aspirin use	3.066 (2.334–4.027)	<0.001
Statin and/or fibrate use	1.664 (1.191–2.325)	0.003
Sulfonyurea use	0.891 (0.664–1.196)	0.443
Metformin use	0.514 (0.382–0.690)	<0.001
Meglitinides use	1.921 (1.393–2.649)	<0.001
Pioglitazone use	0.995 (0.753–1.315)	0.974
Acarbose use	1.683 (1.256–2.255)	<0.001
DPP-4 inhibitor use	0.792 (0.590–1.062)	0.120
Insulin use	2.450 (1.872–3.207)	<0.001

Values expressed as hazard ratios and 95% confidence interval (CI). Abbreviations are the same as in Table 1.

**Table 3:** Determinants for cardiovascular events using the Cox proportional hazards model (multivariable stepwise analysis).

Model	Multivariable (Stepwise)	
	HR (95% CI)	*p*
Model 1		
Age (per 1 year)	1.028 (1.004–1.053)	0.021
Coronary artery disease	3.338 (1.084–10.273)	0.036
Peripheral artery disease	12.362 (1.171–130.56)	0.037
Fasting glucose (per 1 mg/dL)	1.007 (1.005–1.010)	<0.001
eGFR (per 1 mL/min/1.73 m^2^)	0.989 (0.981–0.998)	0.012
UACR (per 10 mg/g)	1.003 (1.001–1.005)	0.004
Anti-hypertensive drugs use	2.185 (1.177–4.056)	0.013
Aspirin use	2.238 (1.418–3.532)	<0.001
Model 2		
Age (per 1 year)	1.040 (1.017–1.065)	<0.001
Coronary artery disease	3.143 (1.024–9.648)	0.045
Stroke	6.189 (1.448–26.457)	0.014
Peripheral artery disease	22.958 (2.317–227.45)	0.007
TyG index (per 1)	1.521 (1.141–2.027)	0.004
UACR (per 10 mg/g)	1.003 (1.002–1.005)	<0.001
Anti-hypertensive drugs use	2.358 (1.272–4.372)	0.007
Aspirin use	2.022 (1.276–3.206)	0.003
Insulin use	2.031 (1.273–3.239)	0.003

Values expressed as hazard ratios (HR) and 95% confidence intervals (CI). Abbreviations are the same as in Table 1. Multivariable stepwise Model 1: adjusted for hemoglobin A_1c_ and significant variables in the univariable analysis except for the TyG index. Multivariable stepwise Model 2: adjusted for hemoglobin A_1c_ and significant variables in the univariable analysis except fasting glucose.

**Table 4:** Determinants for cardiovascular events using the Cox proportional hazards model (multivariable stepwise analysis) after excluding a history of cardiovascular events (*n* = 82).

Model	Multivariable (Stepwise)	
	HR (95% CI)	*p*
Model 1		
Fasting glucose (per 1 mg/dL)	1.003 (1.001–1.005)	0.004
eGFR (per 1 mL/min/1.73 m2)	0.983 (0.978–0.988)	<0.001
Anti-hypertensive drugs use	1.992 (1.378–2.880)	<0.001
Aspirin use	2.382 (1.769–3.209)	<0.001
Model 2		
eGFR (per 1 mL/min/1.73 m2)	0.985 (0.980–0.990)	<0.001
TyG index (per 1)	1.228 (1.015–1.486)	0.035
Anti-hypertensive drugs use	1.911 (1.324–2.759)	<0.001
Aspirin use	1.800 (1.329–2.437)	<0.001
Insulin use	2.306 (1.711–3.107)	<0.001

Values expressed as hazard ratios (HR) and 95% confidence intervals (CI). Abbreviations are the same as in Table 1. Multivariable stepwise Model 1: adjusted for hemoglobin A_1c_ and significant variables in the univariable analysis except for the TyG index. Multivariable stepwise Model 2: adjusted for hemoglobin A_1c_ and significant variables in the univariable analysis except fasting glucose.
